# A non-invasive targeting approach using RK-50 for stereotactic-guided focused ultrasound blood brain barrier opening in mice

**DOI:** 10.1038/s41598-026-61001-y

**Published:** 2026-07-20

**Authors:** Andrew T. Thede, Dylan Borek, Alec J. Batts, Rajiv Chopra, Marc A. Santos, Ibrahim Youssef, Natasha D. Sheybani

**Affiliations:** 1 Department of Biomedical Engineering, University of Virginia, Charlottesville, VA USA; 2 FUS Instruments Inc, Dallas, TX USA; 3 Chemistry Department, Faculty of Science, Mansoura University, Mansoura, Egypt; 4 Department of Radiology & Medical Imaging, University of Virginia, Charlottesville, VA USA; 5 Focused Ultrasound Immuno-Oncology Center, University of Virginia, Charlottesville, VA USA

**Keywords:** Biological techniques, Engineering, Medical research, Neuroscience

## Abstract

Focused ultrasound (FUS) combined with micro/nanobubbles enables transient, localized blood brain barrier opening (BBBO) for targeted drug delivery to the brain. Although imaging-guided FUS methods provide accurate targeting, they require specialized infrastructure that increases cost and workflow complexity. Commercial stereotactic FUS platforms such as the RK-50 offer an accessible alternative, but standard procedures rely on a scalp incision to identify bregma for atlas registration, introducing tissue injury and potential confounds for repeat-BBBO studies. Here, we describe and validate a fully non-invasive targeting approach for FUS BBBO in mice using the RK-50 platform. Our novel targeting method achieved accurate, reproducible targeting without surgical exposure. Evans blue extravasation and atlas co-registration demonstrated sub-millimeter targeting accuracy at bregma and confirmed reliable BBBO in the cortex, hippocampus, and cerebellum. Across these anatomically distinct targets, passive cavitation detection showed broadly comparable acoustic emissions profiles. We further compared this non-invasive workflow with the standard incisional approach in a repeat-BBBO paradigm and observed similar acoustic emissions and comparable delivery of both small molecule and large molecule (antibody) reporters. Together, these findings establish a high-fidelity, incision-free targeting strategy that preserves the accessibility of stereotactic FUS while reducing procedural burden and improving suitability for longitudinal preclinical BBBO studies.

**Supplementary Information:**

The online version contains supplementary material available at https://doi.org/10.1038/s41598-026-61001-y.

## Introduction

Focused ultrasound (FUS) is a burgeoning therapeutic technology for non-invasive, non-ionizing acoustic energy deposition into a target volume, capable of inducing a variety of bioeffects ranging from transient to irreversible. When paired with intravenous administration of gas-filled cavitation nuclei such as nanobubbles (NBs) or microbubbles (MBs), low-intensity pulsed FUS drives intravascular cavitation activity that transiently and reversibly increases BBB permeability by modulating endothelial tight-junction complexes and inducing endothelial sonoporation, thereby enabling both paracellular and transcellular molecular transport^1–3^. The field of FUS-mediated blood brain barrier opening (BBBO) is rapidly expanding, with growing adoption of FUS technology across research groups for applications ranging from molecular payload delivery to neuro- and immunomodulation^4–7^. Ultrasound- or magnetic resonance imaging (MRI)-guided techniques are commonly leveraged to accurately target FUS sonications in a non-invasive manner^8,9^. However, dependence on dedicated imaging infrastructure can increase cost and workflow complexity and may limit accessibility and throughput for routine BBBO studies. The advent of clinical neuronavigation-guided transcranial FUS platforms (e.g. NaviFUS) has accelerated interest in analogous preclinical methods that minimize imaging requirements and streamline experimental workflows^10,11^. Accordingly, research groups worldwide are increasingly leveraging commercial preclinical stereotactic-guided FUS instrumentation (e.g. FUS Instruments RK-50) to enable FUS BBBO with minimal custom hardware development^12–14^.

The RK-50 is a standalone, preclinical transcranial FUS device designed for stereotactic, 3D targeting of the rodent (mouse or rat) brain and supports BBBO with real-time acoustic emissions feedback. Standard operating procedure (SOP) for the RK-50 involves making an incision on the scalp of the subject to identify the bregma suture line. This process aligns the 3D motor system to a brain atlas for accurate stereotactic targeting of the brain with FUS. After FUS treatment, the incision is closed with either surgical glue or sutures. This approach can enable accurate stereotactic targeting without external real-time imaging; however, the need for a scalp incision to enable atlas registration introduces tissue injury (and the ensuing wound-healing/inflammatory response) that can complicate longitudinal/repeat sessions, added procedural burden, and operator-dependent variability. One approach to mitigate these concerns is to use the MRI-based registration and targeting system manufactured by FUS Instruments for the RK-50^15^. But this approach requires access to a preclinical MRI which may be unavailable to many groups. Taken together, these factors motivate the realization of an SOP for a high-fidelity, fully non-invasive targeting approach for stereotactic-guided FUS. In this paper, we provide detailed methods for achieving accurate, non-invasive FUS targeting with the RK-50 (hereafter referred to as NI-FUS) by identifying an alternative fiducial marker through murine scalp.

## Methods

### Animals

Wild-type 8-week-old female C57BL/6 mice from The Jackson Laboratory were used in this study. Mice were housed on a 12/12 h light/dark cycle and supplied food ad libitum. Animal experiments were approved by the Animal Care and Use Committee at the University of Virginia and conformed to the National Institutes of Health guidelines for the use of animals in research. All studies were performed in compliance with ARRIVE guidelines.

### Stereotactic targeting methodology

**Fig. 1:**
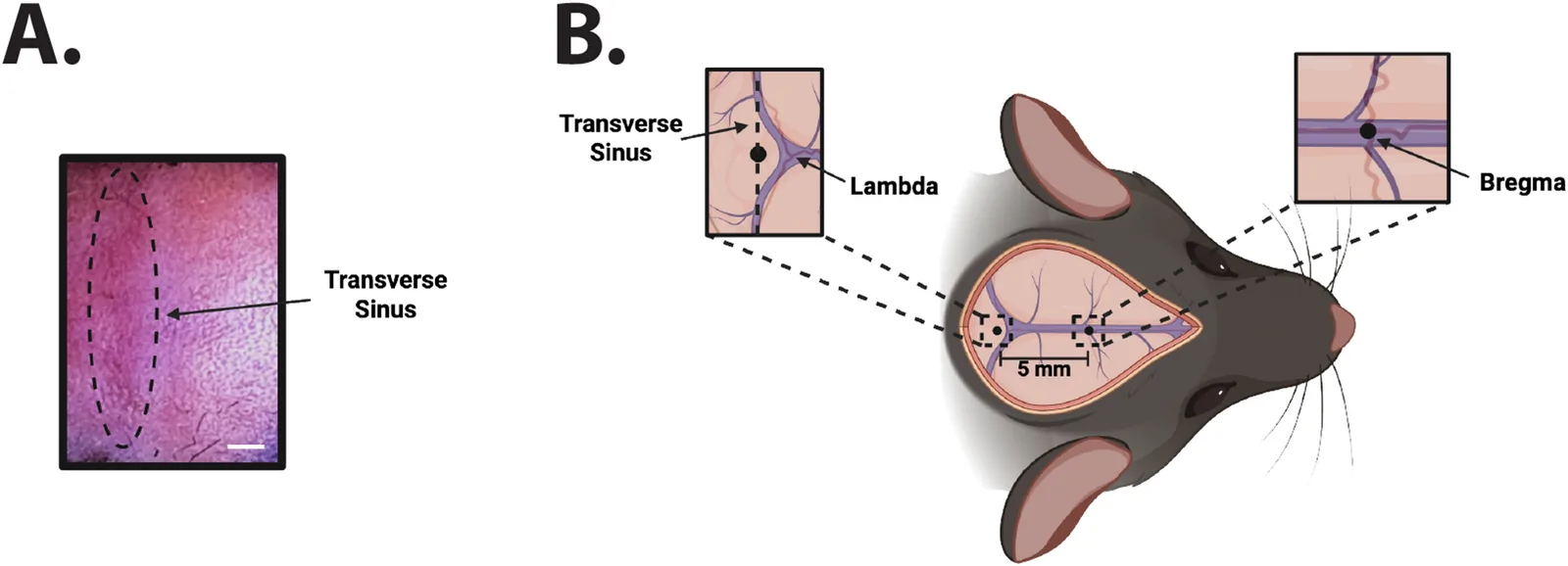
Non-invasive targeting strategy. (**A**) Representative image of transverse sinus visualized on a shaved and depilated mouse scalp. Scale bar indicates 1 mm. (**B**) Schematic of the non-invasive bregma targeting approach, in which the transverse sinus line is visually identified through the intact scalp, and the RK-50 pointer is advanced 5 mm anterior to the transverse sinus line to approximate bregma for atlas registration.

The RK-50 SOP for FUS BBBO relies on the user generating a ~ 0.5–1 cm incision on the scalp of a murine subject while secured on the RK-50 stereotactic platform. Using the RK-50 pointer for focus finding and the RK-50 “Aureus” software, the bregma suture line is identified as an anatomical landmark to reference for accurate targeting of a desired brain region. To non-invasively target bregma for NI-FUS, using the pointer, we first identified and navigated to the transverse sinus line located on the posterior aspect of the scalp, centering the pointer with respect to the head of the mouse (Fig. 1A). Using the RK-50 Aureus software, we subsequently moved the pointer exactly 5 mm anterior to this position to approximate the location of bregma before carefully placing the tip of the apparatus on the scalp to define bregma position on the integrated mouse atlas (Fig. 1B). The choice of 5 mm was justified by direct anatomical measurements as well as corroborating published literature^16^.

### FUS blood brain barrier opening and confirmation

All FUS BBBO procedures were performed on the RK-50. Mice were first anesthetized using 50 mg/kg ketamine and 0.25 mg/kg dexmedetomidine. Heads were depilated and tail veins were cannulated, following which mice were affixed prone to the bite bar and rigid ear bars at fixed, equidistant positions within the RK-50 stereotactic frame. The mouse head positioning was confirmed to be level within the stereotactic frame, ensuring a neutral, non-tilted orientation in the sagittal and coronal planes. A small amount of 70% ethanol was applied to the intact scalp with a cotton swab. We identified the bregma landmark either using the conventional RK-50 SOP or our modified NI-FUS SOP. We applied 10 uL of mineral oil on the scalp of the mouse around the desired target location to minimize the impact of any residual hair-mediated pressure attenuation^17^. We then applied degassed ultrasound gel on the mouse scalp for efficient acoustic coupling. A degassed water-bath separated by a thin layer of Kapton film was subsequently placed in contact with the ultrasound gel, and the FUS transducer was manually lowered into the bath while avoiding formation of air bubbles on the surface of the FUS transducer. Lipid-shelled MBs, formulated as previously described, were administered i.v. in a 50 uL bolus at a dose of 1E5 MBs per gram body weight^18^. FUS BBBO sonications were applied using the “Burst” software setting using a spherically-curved transducer (resonant frequency, f_0_ = 1.0 MHz, f-number = 0.7, aperture diameter = 35 mm, central fenestration for hydrophone insertion = 11 mm) with − 6 dB focal dimensions of 1.38 mm in the lateral direction and 9.8 mm in the axial direction of ultrasound propagation at the focus, measured with a calibrated needle hydrophone (HNA-0400 Needle Hydrophone, Onda Corp., Sunnyvale, CA) in a degassed water tank (Supplementary Fig. 1). The sonication settings were as follows: 10 ms burst length with a pulse repetition period of 2,000 ms for 60 total bursts at a peak-negative-pressure (PNP) of 0.4 MPa.

**Fig. 2:**
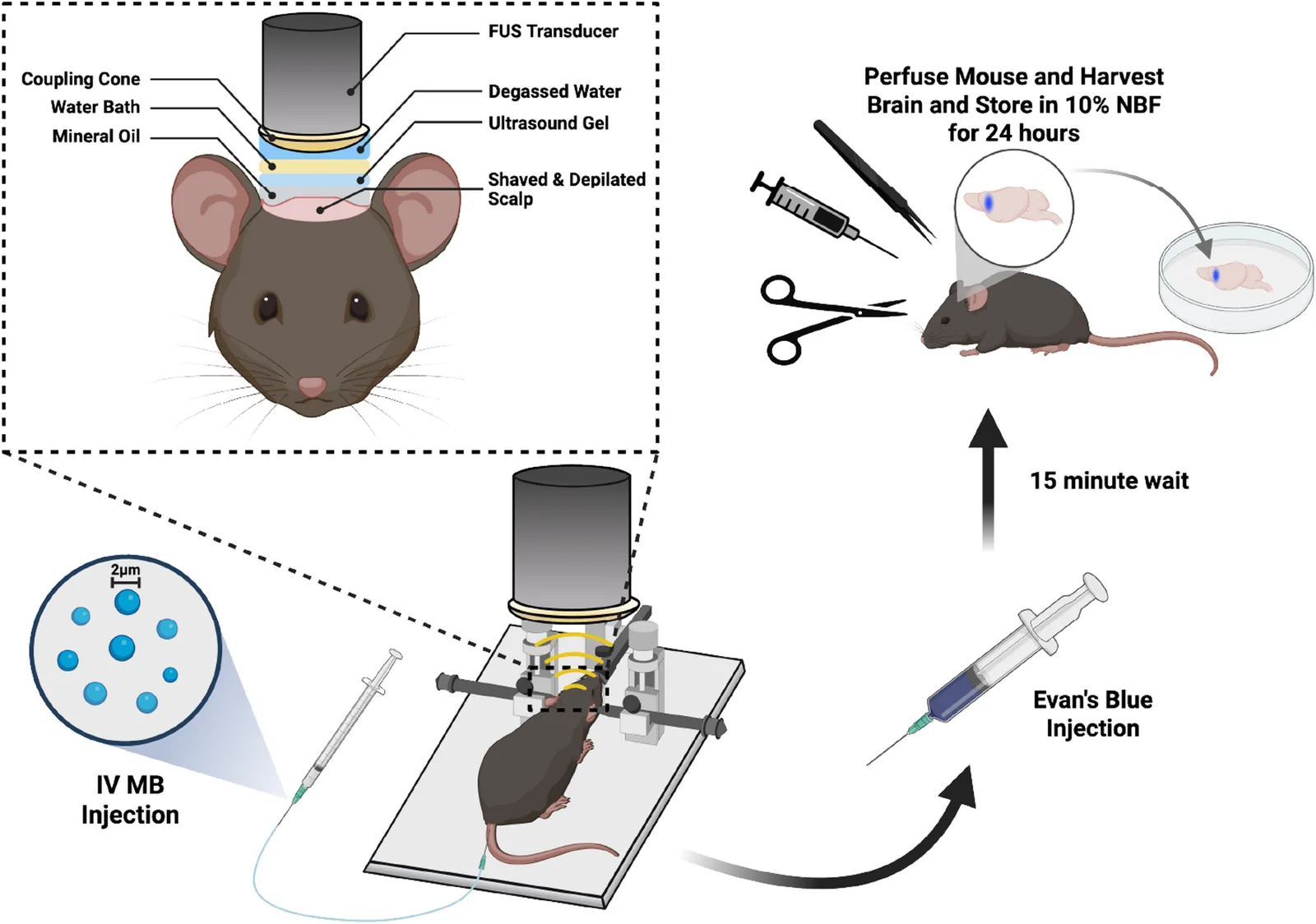
Workflow for RK-50 FUS setup and post-treatment confirmation. Schematic of the experimental setup and treatment workflow for FUS BBBO using the RK-50 stereotactic-guided FUS platform. Mice were positioned on the stereotactic frame with acoustic coupling to the FUS transducer established through mineral oil, ultrasound gel, and a degassed water bath. Following intravenous microbubble administration and sonication, EB was injected systemically to confirm BBB opening. Brains were harvested after a 15-minute circulation period and fixed in 10% neutral buffered formalin for gross assessment of EB extravasation.

Acoustic emissions were monitored with a center-mounted hydrophone embedded within the transducer, consistent with standard RK-50 configurations. Acoustic emissions were filtered with a custom band-pass filter with 0.5 MHz and 5 MHz cutoff frequencies. Prior to MB administration, mice were sonicated using a 10 ms burst length with a pulse repetition period of 2,000 ms for 20 total bursts at a PNP of 0.15 MPa without MBs to acquire spectral baseline data from each mouse for downstream acoustic emissions analysis. Evans Blue (EB) (Sigma-Aldrich, Cat #E2129) extravasation was assessed to confirm FUS BBBO. At the conclusion of sonications, 100 uL of 2 wt% EB dye followed by 30 uL of 2% Heparin-saline, was injected i.v. via the tail canula. Mice were euthanized 15 min post-FUS BBBO, and mice were subsequently exsanguinated through cardiac perfusion with 10 mL of 1X PBS, followed by brain harvest. Brains were stored in 10% neutral buffered formalin for 24 h before generating axially oriented slices for gross macroscopic imaging of EB leakage, ensuring the plane of the brains matches the plane used for stereotactic targeting. The FUS setup and confirmation workflow are summarized in Fig. 2.

### Passive cavitation detection (PCD) analysis

Following FUS treatment, acoustic emissions were processed using a custom, in-house passive cavitation detection (PCD) processing pipeline generated in MATLAB. Acquired voltage data from each RK-50 run was transformed using a Fast-Fourier Transform (FFT) for each FUS burst. We herein define harmonic cavitation dose (HCD), ultraharmonic cavitation dose (UHCD), and inertial cavitation dose (ICD). HCD was calculated by performing a root-mean-square sum (RMS) within a 20 kHz bandwidth centered at the 3rd harmonic (3 MHz) on both baseline and treatment data. The RMS values per burst for the baseline data were subsequently averaged for all bursts to yield an average baseline amplitude value at the 3rd harmonic (3f). For all treatment bursts, data were normalized by dividing each value by the average baseline value for that mouse. The normalized data were subsequently summed to yield a HCD value per target. HCD values per target were averaged to generate the final HCD value per mouse. UHCD was calculated analogously to HCD using the subharmonic (0.5f) and ultraharmonic frequencies (1.5f and 2.5f). We calculated cavitation doses for the subharmonic and ultraharmonic frequency bands independently and subsequently summed these values to yield UHCD. For ICD, RMS was performed on the broadband data bookending a 20 kHz bandwidth centered around either the 1st (1f), 2nd (2f), and 3rd harmonic (3f) or subharmonic (0.5f) and ultraharmonic (1.5f, 2.5f) frequencies (0.51–0.99 MHz; 1.01–1.49 MHz; 1.51–1.99 MHz; 2.01–2.49 MHz; 2.51–2.99 MHz). Data was similarly normalized to the baseline broadband RMS value, summed for all bursts, and averaged for all targets to yield a representative ICD metric per mouse.

### Spatial colocalization analysis

Using a custom algorithm developed in MATLAB, high-resolution images of perfused brain slices bearing EB signal were cropped and scaled such that the true distance per pixel measurement was aligned to the Allen Brain Atlas (Mouse). We then co-localized the FUS targeting strategy within the RK-50 mouse atlas (also based on Allen Brain Atlas) and ex vivo brain images by aligning the boundaries of the left and right striatum between the two images. The opacity of the mouse brain images was decreased by 30% to visualize targeting accuracy. The boundaries of EB signal within the ex vivo brain images were identified using a custom MATLAB script. Using the *WeightedCentroid*() function in MATLAB, we then compared the coordinates of the stereotactic targeting strategy to the coordinates of EB signal to determine targeting accuracy. The difference in distance between centroid of the RK-50 stereotactic target and centroid of ex vivo EB signal is reported herein as a metric of spatial co-localization.

### Ex vivo epifluorescence imaging

The perfused brains of mice receiving either EB or a reporter IgG-AF680 antibody (Invitrogen, Cat #A10038) (administered just prior to sonications) were imaged using the spectral Lago X Imaging System (Spectral Instruments Imaging, Tucson, AZ). EB imaging was performed using an excitation wavelength of 605 nm and an emission wavelength of 690 nm. IgG-AF680 imaging was performed using an excitation wavelength of 675 nm and an emission wavelength of 730 nm. Epifluorescence signal was quantified using the Aura Imaging Software.

### Statistical analysis

All statistical analysis was performed in GraphPad Prism 10 (GraphPad Software, Inc.). Detailed statistics are provided in figure captions. Data are shown as mean +/- SEM. Individual data points reflecting sample sizes are provided in each figure. P-values < 0.05 were considered significant.

## Results

### Non-invasive bregma targeting achieves sub-millimeter spatial accuracy

**Fig. 3:**
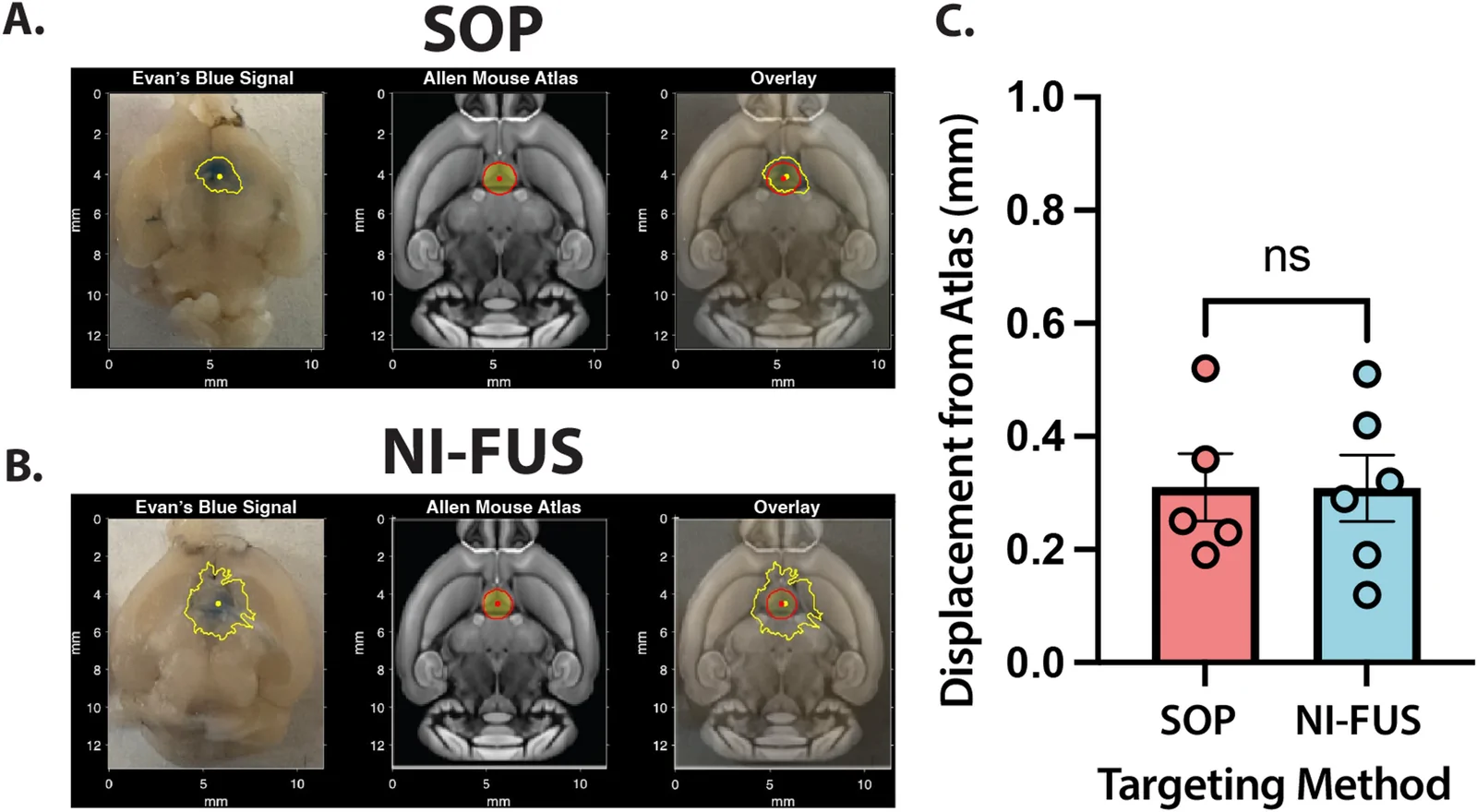
Spatial accuracy assessment of NI-FUS. (**A**,**B**) Representative Evans blue signal, corresponding Allen Mouse Brain Atlas target, and overlay demonstrating localization of BBBO at bregma using (**A**) RK-50 SOP approach and (**B**) NI-FUS approach. Yellow outlines denote EB extravasation, and the red circles indicate the planned target. (C) Centroid-to-centroid distances between planned and observed target locations across individual brains, demonstrating sub-millimeter targeting accuracy and minimal statistical variation between targeting approaches. Statistical significance assessed via two-tailed unpaired Student’s T-Test (SOP *N* = 5; NI-FUS *N* = 6), *p < 0.05.

To establish whether bregma could be targeted without surgical scalp exposure, we first applied both the RK-50 SOP and the NI-FUS workflow to a single sonication point centered at the atlas origin. EB extravasation on post-treatment brain sections, together with co-registration to the Allen Mouse Brain Atlas, demonstrated accurate localization of BBB opening at the intended bregma target for both SOP and NI-FUS approaches (Fig. 3A-B). Quantitative centroid analysis confirmed sub-millimeter spatial accuracy, with a mean displacement of 0.31 mm between the planned target and observed EB signal for both techniques (Fig. 3C). These findings indicate that non-invasive landmarking provides sufficient precision for stereotactic FUS BBBO using the RK-50 platform, identical to that of the SOP landmarking approach.

### Non-invasive targeting supports accurate FUS BBBO across anatomically distinct brain regions

**Fig. 4:**
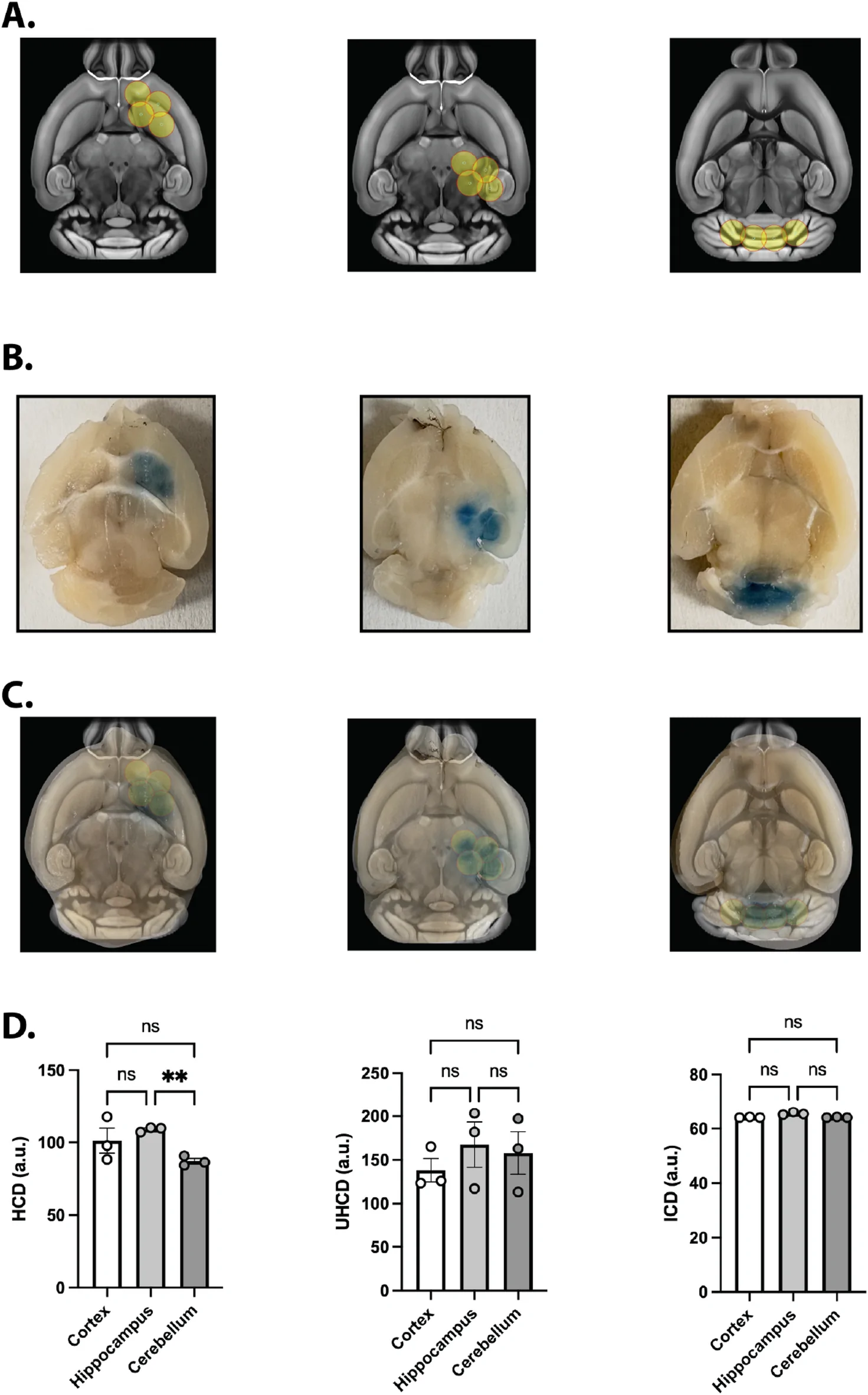
Non-invasive stereotactic targeting supports accurate FUS BBBO across multiple brain regions with comparable cavitation activity. (**A**) Planned targeting schemes for the cortex, hippocampus, and cerebellum based on the Allen Mouse Brain Atlas. (**B**) Representative axial brain slices showing EB extravasation following FUS BBBO in each target region. (**C**) Overlays of ex vivo brain images with atlas-defined target locations demonstrating spatial concordance between planned and observed BBB opening. (**D**) Harmonic cavitation dose (HCD), ultraharmonic cavitation dose (UHCD), and inertial cavitation dose (ICD) across targeted brain regions measured via real-time passive cavitation detection. Together, these data show that the non-invasive targeting strategy reliably localizes BBBO to anatomically distinct regions while yielding broadly comparable acoustic emissions profiles. Significance assessed by one-way ANOVA with Tukey’s post-hoc test (Cortex *N* = 3, Hippocampus *N* = 3, Cerebellum *N*=3), *p < 0.05, **p < 0.01.

We next asked whether this non-invasive targeting strategy could be generalized beyond bregma to brain regions commonly interrogated in preclinical FUS studies^13,19,20^. Using atlas-defined coordinates, we targeted the cortex, hippocampus, and cerebellum (Fig. 4A). In each case, post-treatment brain sections showed localized EB extravasation at the intended site of sonication and overlay with atlas-defined targets demonstrated strong spatial concordance between planned and observed BBB opening (Fig. 4B-C). Together, these data show that the non-invasive workflow is not limited to a single fiducial point but can reliably support targeting of multiple anatomically distinct regions.

We then compared passive cavitation signatures across these targets to determine whether regional targeting influenced microbubble activity. Harmonic cavitation dose was similar in the cortex and hippocampus and modestly lower in the cerebellum, whereas ultraharmonic and inertial cavitation doses were broadly comparable across all three regions (Fig. 4D). These findings suggest that the non-invasive targeting approach yields consistent cavitation behavior across brain regions, with only modest regional variation in harmonic emissions.

### Non-invasive targeting yields repeat-session cavitation and model drug delivery comparable to the standard incisional workflow

**Fig. 5:**
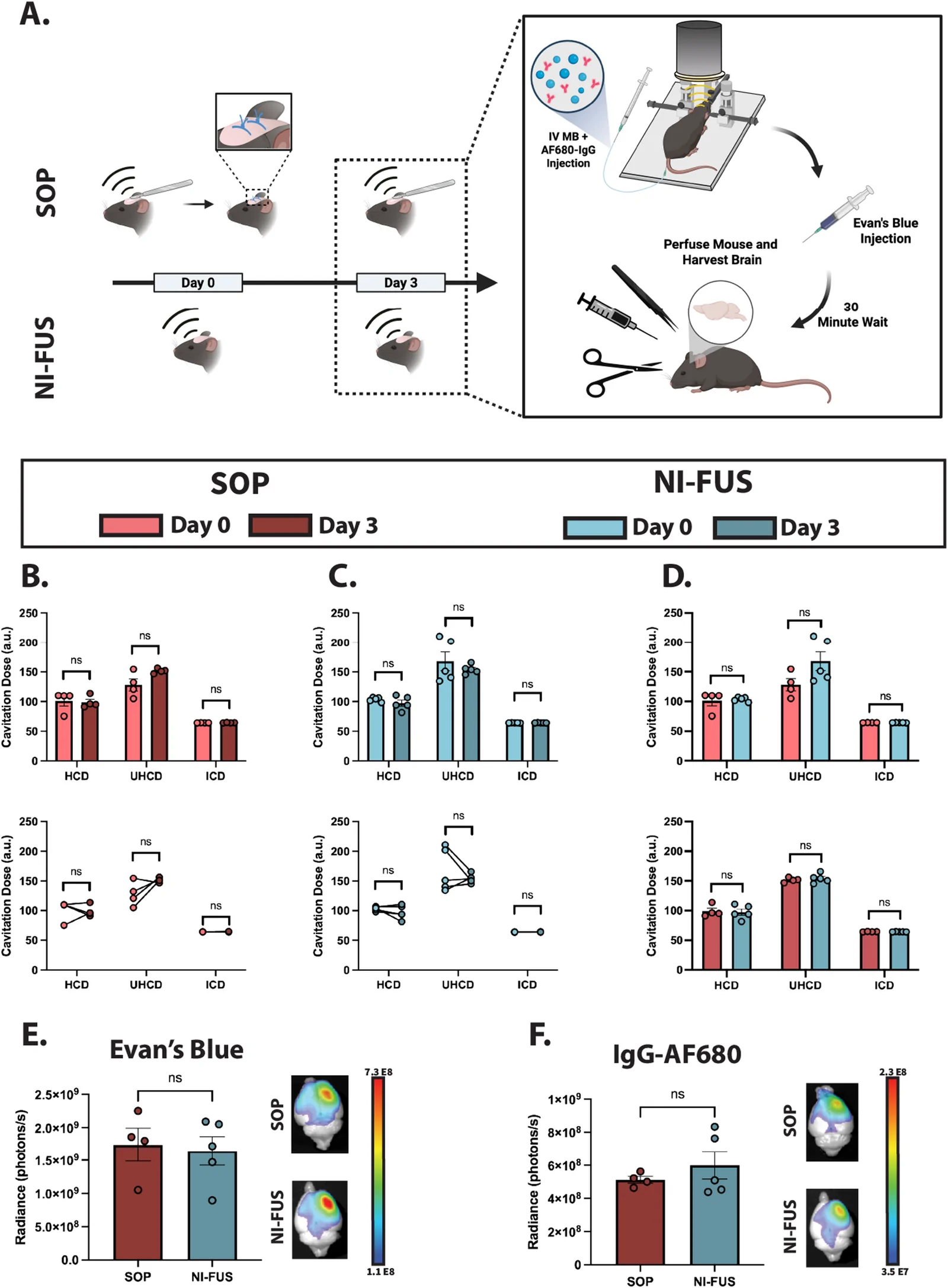
Non-invasive targeting yields repeat-session cavitation and model drug delivery comparable to the standard incisional RK-50 workflow. (**A**) Experimental design comparing the conventional incisional targeting method and the incisionless targeting method across two FUS BBBO sessions separated by 3 days. On the second treatment day, AF680-labeled IgG was administered before sonication and Evans blue was administered after treatment before perfusion and brain harvest. (**B**–**D**) Comparisons of harmonic, ultraharmonic, and inertial cavitation dose across treatment days (B, C) and targeting approaches (D), showing similar acoustic activity between workflows. (**E**) Quantification (left) and representative ex vivo fluorescence images (right) of Evans blue delivery to the brain between incisional and incisionless targeting approaches. (**F**) Quantification (left) and representative ex vivo fluorescence images (right) of IgG-AF680 delivery to the brain between incisional and incisionless targeting approaches. Significance assessed by two-tailed paired T-test and two-tailed unpaired T-test where appropriate (SOP *N* = 4, NI-FUS *N* = 5), *p < 0.05.

Having established targeting accuracy, we next evaluated whether the non-invasive workflow performs comparably to the standard RK-50 incisional approach in a repeated-treatment setting. Mice underwent two FUS BBBO sessions separated by 3 days using either the SOP or NI-FUS targeting method (Fig. 5A). To assess delivery across molecular size scales, AF680-labeled IgG was administered before the second sonication and EB was administered immediately after treatment prior to tissue collection. Across both treatment sessions, passive cavitation analysis demonstrated comparable harmonic, ultraharmonic, and inertial emissions between workflows (Fig. 5B-D), indicating that elimination of the scalp incision does not adversely alter acoustic activity during BBBO. Consistent with these findings, ex vivo fluorescence imaging showed similar brain delivery of both EB and IgG-AF680 between the incisional and non-invasive groups (Fig. 5E-F). Collectively, these data indicate that the non-invasive RK-50 targeting method preserves both cavitation performance and model drug delivery while avoiding the procedural burden of surgical landmark exposure.

## Discussion

In this study, we established and validated a fully non-invasive targeting workflow for stereotactic-guided FUS-mediated BBB opening in mice using the RK-50 platform. By identifying the transverse sinus line through the intact scalp and advancing 5 mm anteriorly to approximate bregma, we achieved accurate and reproducible atlas registration without surgical scalp exposure. This approach yielded sub-millimeter targeting accuracy at bregma, analogous to the SOP approach for the RK-50. NI-FUS also supported reliable BBB opening across multiple anatomically distinct brain regions, including the cortex, hippocampus, and cerebellum. Importantly, the NI-FUS workflow also enabled scalable repeat FUS sessions, with cavitation activity and model drug delivery comparable to the standard incisional RK-50 procedure. Collectively, these findings support this incision-free strategy as a practical and high-fidelity alternative for preclinical FUS BBBO studies.

A key finding of this work is that accurate stereotactic targeting can be achieved without external image guidance or surgical landmark exposure. The observed mean targeting displacement of 0.31 mm falls well within the lateral focal dimensions of the 1 MHz transducer used here (1.38 mm), indicating that the spatial precision of this approach is appropriate for routine murine FUS BBBO applications. Our use of a 1 MHz FUS transducer here is noteworthy with respect to these accuracy results. When operating focused transducers at higher frequencies, the lateral dimension of the focus will decrease. We recommend particular care when employing NI-FUS at these acoustic exposure conditions. Yet given the accuracy of our measurements was determined from the center of the EB signal, we anticipate similar degrees of accuracy regardless of transducer frequency. More broadly, these data suggest that minor operator-dependent variability inherent to stereotactic workflows does not preclude reliable BBB opening when anatomical registration is performed through intact scalp landmarking. This is particularly relevant for laboratories seeking accessible, streamlined alternatives to MRI- or ultrasound-guided targeting workflows.

Beyond accuracy, the non-invasive nature of this method offers practical and biological advantages. Standard RK-50 operation requires a scalp incision to expose bregma, followed by wound closure and, in repeat-session studies, re-opening of the surgical site. By eliminating this step, the workflow reduces procedural burden, simplifies repeat treatments, and better aligns with the inherently non-invasive premise of FUS BBBO. This feature may be especially valuable in study designs involving serial sonications over short intervals, where incomplete wound healing after repeated scalp incision could introduce experimental variability and compromised acoustic transmission. Incision-associated tissue injury may also confound interpretation of treatment-induced biological effects, particularly in studies interrogating neuroinflammation, immune activation, or therapeutic response^21^.

Notably, this workflow generalized well across multiple brain regions. Passive cavitation analysis demonstrated broadly comparable harmonic, ultraharmonic and inertial emissions across the cortex, hippocampus, and cerebellum, with only a modest reduction in harmonic emissions in the cerebellar target. One possible explanation is geometric rather than methodological: cerebellar targeting required adjustment of the focal position to avoid excessive exposure of the brainstem, which may have altered the effective tissue volume encompassed by the focal region. In addition, skull curvature and angle of incidence likely vary across these anatomical locations and may modestly influence acoustic transmission. Importantly, despite these differences in harmonic emissions, robust Evans blue extravasation was still achieved in the cerebellum, indicating that effective BBB opening was preserved.

The repeat-session comparison further highlights the translational relevance of the non-invasive approach. Across two treatments separated by 3 days, incision-free targeting produced cavitation profiles comparable to those observed with the standard incisional workflow and supported similar delivery of both Evans blue (~ 0.96 kDa) and reporter IgG (~ 150 kDa). These findings indicate that eliminating surgical landmark exposure does not compromise acoustic performance or brain delivery of either small- or large-molecule reporters in a naïve setting. This is an important point for preclinical drug-delivery studies, where the RK-50 is commonly used to evaluate FUS-enhanced delivery of therapeutic agents to the brain. The ability to maintain delivery performance while removing a surgically induced variable may be particularly advantageous in longitudinal designs and in studies where inflammatory or wound-healing responses could otherwise obscure interpretation.

This study does have limitations. First, validation was performed in healthy, 8-week-old female C57BL/6 mice, and future work should determine how well this targeting strategy generalizes across strains, ages, sexes, and disease models. Past work has investigated the skull morphological differences between mice of different strains, finding some mouse strain-related variations in skull anatomy^22^. We performed these studies in the most widely used mouse strain (C57BL/6) to maximize applicability of our NI-FUS SOP to adopters^23^. The NI-FUS workflow, although validated in C57BL/6, can be applied to other mouse strains following similar validation between transverse sinus and bregma structures. Previous studies have demonstrated that murine skull shape and size remain consistent ~ 60 days after birth^24^. Moreover, rigorous comparisons of bregma-lambda distances between young (2–3 months) and old (23–29 months) male and female C57BL/6 mice have found distances to be very similar irrespective of sex or age^25^. Although mature murine skull anatomy has been shown to be relatively stable after early postnatal development, pathological settings such as intracranial tumors, edema, or stroke could alter tissue geometry and BBB status in ways that influence targeting or delivery. Second, we evaluated delivery using model small- and large-molecule tracers rather than active therapeutics, and we limited repeat-session testing to an acute two-treatment paradigm. Future studies in orthotopic disease models and in extended longitudinal regimens will be useful to define the full operating range and biological advantages of this workflow.

Overall, these findings provide a simple and accessible method for non-invasive, stereotactic-guided FUS BBBO using the RK-50 platform. By preserving targeting accuracy and delivery performance while avoiding surgical landmark exposure, this approach lowers practical barriers to repeated stereotactic FUS studies and may reduce incision-associated confounds in experiments designed to interrogate biological response. More broadly, this work advances a procedural framework that is better aligned with the non-invasive goals of BBB opening paradigms and should be readily adaptable across preclinical laboratories using stereotactic FUS systems.

## Supplementary Information

Below is the link to the electronic supplementary material.

Supplementary Material 1

## Data Availability

All data generated or analysed during this study are included in this published article.

## References

[1] Gorick, C. M. et al. Applications of focused ultrasound-mediated blood-brain barrier opening. *Adv. Drug Deliv. Rev.* **191** (2022). 10.1016/j.addr.2022.114583 PMC9712235 36272635

[2] Zhu, H., Allwin, C., Bassous, M. G. & Pouliopoulos, A. N. Focused ultrasound-mediated enhancement of blood–brain barrier permeability for brain tumor treatment: a systematic review of clinical trials. *J. Neurooncol*. **170**, 235–252 (2024). 10.1007/s11060-024-04795-z PMC11538134 39207625

[3] Chen, K. T., Wei, K. C. & Liu, H. L. Focused Ultrasound Combined with Microbubbles in Central Nervous System Applications. *Pharmaceutics* **13**, 1084 (2021). 10.3390/pharmaceutics13071084 PMC8308978 34371774

[4] Meng, Y., Hynynen, K. & Lipsman, N. Applications of focused ultrasound in the brain: from thermoablation to drug delivery. *Nat. Rev. Neurol.* **17**, 7–22 (2021). 10.1038/s41582-020-00418-z 33106619

[5] Park, E. J., Zhang, Y. Z., Vykhodtseva, N. & McDannold, N. Ultrasound-mediated blood-brain/blood-tumor barrier disruption improves outcomes with trastuzumab in a breast cancer brain metastasis model. *J. Controlled Release*. **163**, 277–284 (2012). 10.1016/j.jconrel.2012.09.007 PMC3502612 23000189

[6] Jeong, J. et al. Aducanumab delivery via focused ultrasound-induced transient blood–brain barrier opening in vivo. *Sci. Rep.* **15**, 17742 (2025). 10.1038/s41598-025-02412-1 PMC12098756 40404732

[7] Li, W. et al. Precise antibody delivery to the brain via nanobubble-actuated focused ultrasound alleviates depression. *Proc. Natl. Acad. Sci.* **122**, e2421800122 (2025). 10.1073/pnas.2421800122 PMC12415236 40864654

[8] Anastasiadis, P. et al. Localized blood-brain barrier opening in infiltrating gliomas with MRI-guided acoustic emissions-controlled focused ultrasound. *Proc. Natl. Acad. Sci. U. S. A.* **118**, e2103280118 (2021). 10.1073/pnas.2103280118 PMC8449371 34504017

[9] Batts, A. J. et al. Using a novel rapid alternating steering angles pulse sequence to evaluate the impact of theranostic ultrasound-mediated ultra-short pulse length on blood-brain barrier opening volume and closure, cavitation mapping, drug delivery feasibility, and safety. *Theranostics* **13**, 1180–1197 (2023). 10.7150/thno.76199 PMC9925313 36793858

[10] Chen, K. T. et al. Neuronavigation-guided focused ultrasound (NaviFUS) for transcranial blood-brain barrier opening in recurrent glioblastoma patients: clinical trial protocol. *Ann. Transl Med.* **8**, 673 (2020). 10.21037/atm-20-344 PMC7327352 32617293

[11] Wu, C. C. et al. Blood-brain barrier opening with neuronavigation-guided focused ultrasound in pediatric patients with diffuse midline glioma. *Sci. Transl Med.* **17**, eadq6645 (2025). 10.1126/scitranslmed.adq6645 41223245

[12] Uribe Cardenas, R. et al. Influence of focused ultrasound on locoregional drug delivery to the brain: Potential implications for brain tumor therapy. *J. Controlled Release*. **362**, 755–763 (2023). 10.1016/j.jconrel.2023.08.060 37659767

[13] Martinez, P., Bottenus, N. & Borden, M. Cavitation Characterization of Size-Isolated Microbubbles in a Vessel Phantom Using Focused Ultrasound. *Pharmaceutics* **14**, 1925 (2022). 10.3390/pharmaceutics14091925 PMC9501432 36145673

[14] Lapin, N. A., Gill, K., Shah, B. R. & Chopra, R. Consistent opening of the blood brain barrier using focused ultrasound with constant intravenous infusion of microbubble agent. *Sci. Rep.* **10**, 16546 (2020). 10.1038/s41598-020-73312-9 PMC7538995 33024157

[15] Martinez, P. et al. MRI-guided focused ultrasound blood–brain barrier opening increases drug delivery and efficacy in a diffuse midline glioma mouse model. *Neuro-Oncol Adv.* **5**, vdad111 (2023). 10.1093/noajnl/vdad111 PMC10547466 37795179

[16] Jun, H., Chavez, J., Bramian, A. & Igarashi, K. M. Protocol for remapping of place cells in disease mouse models. *STAR. Protoc.* **2**, 100759 (2021). 10.1016/j.xpro.2021.100759 PMC8384906 34467228

[17] Xu, L., Gong, Y., Chien, C. Y., Leuthardt, E. & Chen, H. Transcranial focused ultrasound-induced blood–brain barrier opening in mice without shaving hairs. *Sci. Rep.* **13**, 13500 (2023). 10.1038/s41598-023-40598-4 PMC10439893 37598243

[18] Burgess, A., Nhan, T., Moffatt, C., Klibanov, A. L. & Hynynen, K. Analysis of focused ultrasound-induced blood–brain barrier permeability in a mouse model of Alzheimer’s disease using two-photon microscopy. *J. Controlled Release*. **192**, 243–248 (2014). 10.1016/j.jconrel.2014.07.051 25107692

[19] Nouraein, S. et al. Acoustically targeted noninvasive gene therapy in large brain volumes. *Gene Ther.* **31**, 85–94 (2024). 10.1038/s41434-023-00421-1 37696982

[20] Sheybani, N. D. et al. ImmunoPET-informed sequence for focused ultrasound-targeted mCD47 blockade controls glioma. *J. Control. Release.* **10**, 19-29 (2021). 10.1016/j.jconrel.2021.01.023 PMC7946780 33476735

[21] McMahon, D., Bendayan, R. & Hynynen, K. Acute effects of focused ultrasound-induced increases in blood-brain barrier permeability on rat microvascular transcriptome. *Sci. Rep.* **7**, 45657 (2017). 10.1038/srep45657 PMC5379491 28374753

[22] Kawakami, M. & Yamamura, K. Cranial bone morphometric study among mouse strains. *BMC Evol. Biol.* **8**, 73 (2008). 10.1186/1471-2148-8-73 PMC2287174 18307817

[23] Bryant, C. D. The blessings and curses of C57BL/6 substrains in mouse genetic studies. *Ann. N Y Acad. Sci.* **1245**, 31–33 (2011). 10.1111/j.1749-6632.2011.06325.x PMC4944652 22211972

[24] Maga, A. M. Postnatal Development of the Craniofacial Skeleton in Male C57BL/6J Mice. *J. Am. Assoc. Lab. Anim. Sci. JAALAS*. **55**, 131–136 (2016). PMC4783629 27025802

[25] Knight, Z. A. & Jang, H. Age-related morphological changes in the C57BL/6J mouse calvaria: implications for stereotaxic neurosurgery. *bioRxiv. *10.64898/2026.04.24.720718 (2026).

