# Supplementary material for: A non-invasive targeting approach using RK-50 for stereotactic-guided focused ultrasound blood brain barrier opening in mice
Source: Sci Rep. 2026 Jul 20;16:33115. doi: 10.1038/s41598-026-61001-y (PMC13683041; doi:10.1038/s41598-026-61001-y)
Supplement: Supplementary file 1 [file 41598_2026_61001_MOESM1_ESM.pdf]

Supplemental Materials For:

A Non-Invasive Targeting Approach Using RK-50 for Stereotactic-Guided Focused Ultrasound Blood Brain Barrier Opening in Mice

Andrew T. Thede[1], Dylan Borek[1], Alec J. Batts, Ph.D.[1], Rajiv Chopra, Ph.D.[2], Marc A. Santos, Ph.D.[2], Ibrahim Youssef, Ph.D.[2,3], Natasha D. Sheybani, Ph.D.[1,4,5,*]

[1]Department of Biomedical Engineering, University of Virginia, Charlottesville, VA, USA

[2]FUS Instruments Inc., Dallas, TX, USA

[3]Chemistry Department, Faculty of Science, Mansoura University, Egypt

[4]Department of Radiology & Medical Imaging, University of Virginia, Charlottesville, VA, USA

[5]Focused Ultrasound Immuno-Oncology Center, University of Virginia, Charlottesville, VA, USA

***Corresponding Author:**

Natasha D. Sheybani, Ph.D.

Department of Biomedical Engineering

University of Virginia Health System

Box 800759

Charlottesville, VA 22908, USA

Phone: 434-982-4269

Email: nds3sa@virginia.edu

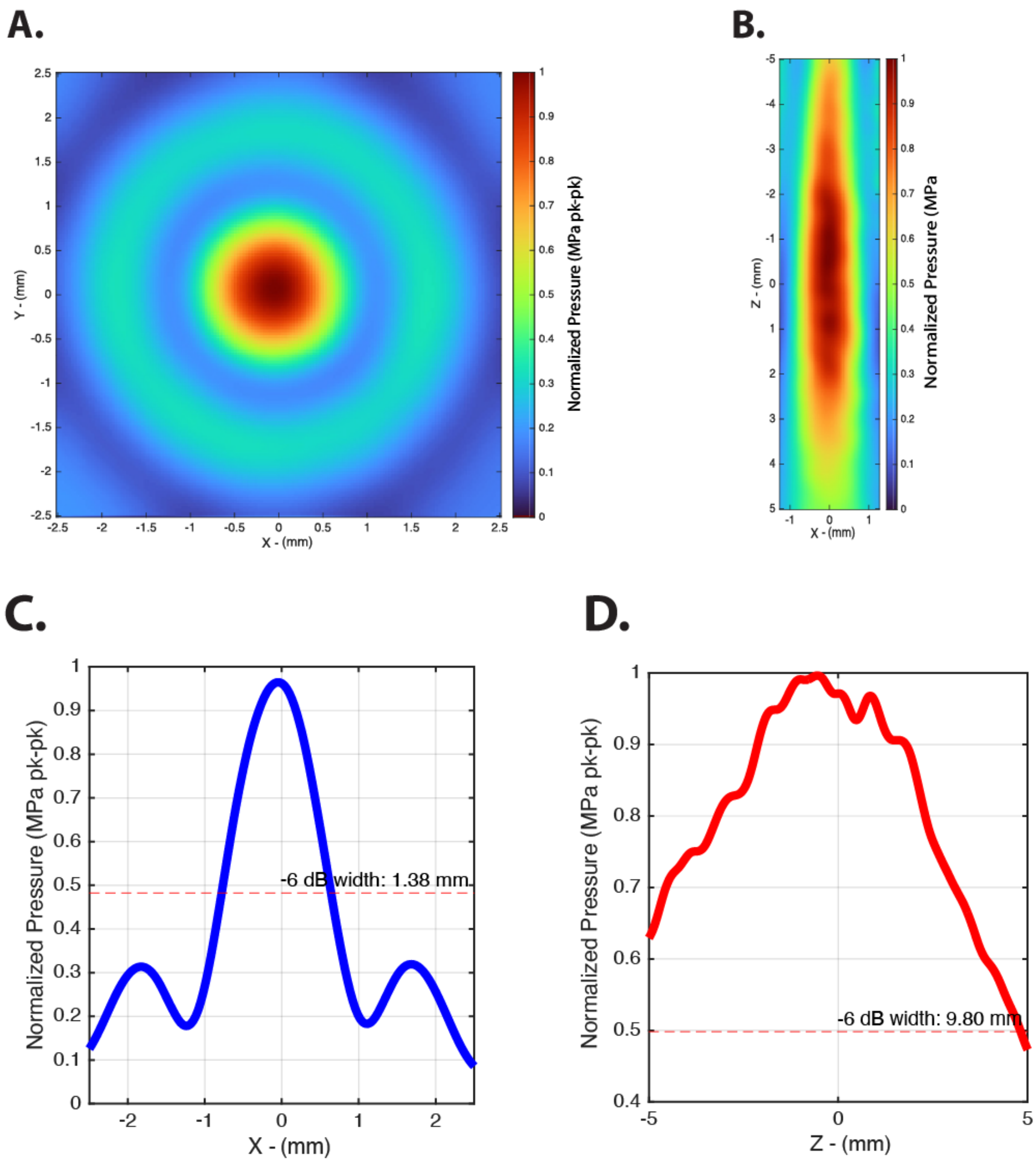

Supplementary Figure 1. Characterization of 1 MHz Focused Ultrasound Transducer. (A) Lateral raster scan of 1 MHz transducer used in the study. (B) Axial raster scan of 1 MHz transducer used in the study. (C) Raster scan profile of lateral raster scan. Full-Width-Half-Max (FWHM) determined at -6 dB. (D) Raster scan profile of axial raster scan. FWHM determined at -6 dB. Acoustic characterization measurements made with HNA-0400 Needle Hydrophone. A median filter was applied to scan maps to reduce motion-attributed jitter artifacts in MATLAB.
